# Effect study of exosomes derived from platelet-rich plasma in the treatment of knee cartilage defects in rats

**DOI:** 10.1186/s13018-023-03576-0

**Published:** 2023-03-02

**Authors:** Hangyu Zhao, Zihang Zhao, Dailuo Li, Xin Wang, Dehao Dai, Hailiang Fu

**Affiliations:** 1 Department of Orthopedics, The Second Affiliated Hospital of Harbin Medical University, No. 246, Xuefu Road, Nangang District, Harbin, 150001 Heilongjiang China; 2 Department of Orthopedics, Chengdu Seventh People’s Hospital, No. 1188, Shuangxing Avenue, Shuangliu District, Chengdu, 610044 Sichuan China

**Keywords:** Platelet-rich plasma, Exosome, Cartilage defect, Cartilage repair

## Abstract

**Background:**

The repair of articular cartilage defects has always been a difficult problem. We aimed to investigate the therapeutic effect of intra-articular injection of platelet-rich plasma (RPR) and PRP-derived exosomes (PRP-Exos) on cartilage defects in rat knee joints and then provide experience for the use of PRP-exos in cartilage defect repair.

**Methods:**

Rat abdominal aortic blood was collected, and PRP was extracted by two-step centrifugation. PRP-exos were obtained by kit extraction, and PRP-exos were identified by various methods. After the rats were anesthetized, a cartilage defect subchondral bone was created at the proximal end of the origin of the femoral cruciate ligament with a drill. SD rats were divided into 4 groups, including PRP group, 50 μg/ml PRP-exos group, 5 μg/ml PRP-exos group, and control group. One week after the operation, 50 μg/ml PRP, 50 μg/ml PRP-exos, 5 μg/ml PRP-exos and normal saline were injected into the knee joint cavity of rats in each group, once a week. A total of two injections were given. On the 5th and 10th week after drug injection, the serum levels of matrix metalloproteinase 3 (MMP-3) and tissue inhibitor of matrix metalloproteinase 1 (TIMP-1) were detected by each treatment method, respectively. The rats were killed at the 5th and 10th weeks, respectively, and the cartilage defect repair was observed and scored. The defect repair tissue sections were used for HE staining and type II collagen immunohistochemical staining.

**Results:**

The histological results showed that both PRP-exos and PRP could promote cartilage defect repair and type II collagen formation, and the promoting effect of PRP-exos was significantly better than that of PRP. In addition, enzyme-linked immunosorbent assay (ELISA) results showed that compared with PRP, PRP-exos could significantly increase serum TIMP-1 and decrease serum MMP-3 in rats. And the promoting effect of PRP-exos was concentration dependent.

**Conclusion:**

Intra-articular injection of PRP-exos and PRP can promote the repair of articular cartilage defects, and the therapeutic effect of PRP-exos is better than the same concentration of PRP. PRP-exos are expected to be an effective treatment for cartilage repair and regeneration.

## Introduction

In recent decades, cartilage injury-related diseases have become more and more common in clinical practice. Sports-induced injuries, acute trauma and trauma, arthritis, and joint bacterial infections can all lead to cartilage damage and cartilage tissue loss [1]. It is difficult to repair and treat cartilage damage. Current treatment methods include chondrocyte transplantation, cartilage transplantation, microfracture stimulation therapy, osteotomy, etc. These treatment methods can prevent further joint degeneration, but cannot completely repair damaged cartilage. For example, microfracture treatment can damage the subchondral bone, causing endochondral osteophytes, while osteotomy can leave lifelong pain and discomfort [2, 3].

Exosomes are active vesicles with bilayer lipid membranes, ranging in diameter from 30–100 nm to 40–200 nm, containing a variety of active substances such as proteins, lipids, mRNAs, and miRNAs [4]. Exosomes can mediate biological function transmission and intercellular communication [5].

Exosomes evolve from early endosomes in cells. The vesicle membrane of endosomes is inwardly recessed. After maturity, they become intracellular polycystic bodies, and MVBs are released by fusion with the cytoplasmic membrane [6]. Under physiological conditions, almost all metabolically active cells can release exosomes and transport them to various parts of the body through the blood, lymph, cerebrospinal fluid and other body fluid systems. Studies have shown that exosomes can be found in blood, saliva, cerebrospinal fluid, breast milk, urine, etc. [7–9]. There is solid evidence that exosomes have similar functional roles to the cells from which they are derived, and stress conditions such as tissue damage and hypoxia can affect the composition, synthesis, and secretion of exosomes in relevant cells [10–12]. In recent years, many studies have shown that exosomes play an important role in antiviral, regenerative medicine and other fields [9, 13]. This shows that exosomes have promising research prospects.

Platelets are non-nucleated cells without genomic DNA, but they contain abundant and diverse mRNAs and miRNAs, and are the main source of exosomes in human circulating plasma [14, 15]. It has even been reported in the literature that platelet-derived exosomes account for up to 70% of the total serum exosomes and are involved in various important pathophysiological mechanisms such as inflammation and atherosclerosis [16]. PRP-exos are platelet-derived exosomes extracted from PRP by various methods such as filtration and centrifugation. Its size, shape and some protein markers are similar to exosomes from other cells. It is also the main mediator of intercellular communication. The difference is that platelet exosomes contain more chemokines, growth factors, and other active substances [17].

PRP is a multifunctional platelet concentrate obtained by repeated centrifugation of animal or human whole blood, and its platelet concentration is always higher than that of peripheral blood [18, 19]. The ingredients include high concentrations of various growth factors, fibrin, and white blood cells [20].

PRP has the functions of promoting cell proliferation and differentiation, extracellular matrix generation, and anti-inflammatory. It has been widely used in the regeneration and repair of bone tissue, cartilage tissue, tendon, ligament, skin defect, nerve injury, beauty, and hair growth. The mechanism of PRP promoting cartilage repair is mainly tissue regeneration mediated by various growth factors and inhibition of matrix metalloproteinases to protect cartilage tissue. Exosomes may be the main way for PRP to play a repair role [21, 22]. It has been reported that the tissue repair activity of PRP may be due to the efficient intercellular communication of growth factors and other bioactive molecules, which are mediated by piggybacking on PRP-exos [23]. In the study of Guo et al., PRP-exos played a good role in tissue repair [24, 25].

This study aims to explore the repairing effect of PRP-exos on articular cartilage defects by manufacturing articular cartilage defects and articular cavity injection treatment, comparing it with the cartilage repairing effect of PRP, and exploring the repairing effect of PRP-exos at different concentrations. The impact of PRP-exos provides experience for the clinical treatment of articular cartilage repair.

## Material and methods

### Experimental animals

Female SD rats (n = 38, 10-week-old, 280–320 g) were purchased from the Animal Experiment Center of the Second Affiliated Hospital of Harbin Medical University. All the animal experiments were authorized by Harbin Medical University's Animal Use and Care Committee (approval number: SYDW 2020–020) and were conducted in compliance with US NIH criteria. Three rats provided whole blood for exosome extraction and 3 rats provided whole blood for PRP extraction.

### PRP extraction

Intraperitoneal injection of 3% pentobarbital sodium solution (injection dose: 1.0 ml/kg), after anesthesia is completed, the abdominal skin was disinfected, skin prepared, the abdominal skin was cut, the abdominal aorta was separated and exposed, the distal end of the blood vessel was clamped, and the end of the blood collection needle connected the anticoagulant tube for blood collection, collect about 9–10 ml of whole blood. The anticoagulant tube contains the anticoagulant citric acid glucose solution A, with a ratio of 9:1 (v/v). Then, we fully shook the blood in the anticoagulant tube and set aside 0.2 ml for platelet count detection. According to the Landesberg two-step centrifugation method [26], the whole blood was centrifuged at 200×*g* for 10 min at 4 °C, the whole blood is divided into two layers in the centrifuge tube. Then, we absorbed all the upper plasma and continued to centrifuge at 200×*g* for 10 min, red blood cells were precipitated at the bottom of the tube, and the lower 1/4 of the remaining plasma was PRP, about 1.6–1.8 ml, took 0.2 ml for platelet count detection, and temporarily store the remaining PRP at 4 °C. In subsequent experiments, we used non-activated PRP.

### PRP-exosomes extraction

We distributed 1–1.5 ml of PRP into 1.5 ml centrifuge tubes and extracted it according to the instructions of Total Exosome Isolation (from plasma) (Cat. no.4484450, Invitrogen). At room temperature, the supernatant was aspirated after centrifugation at 2000×*g* for 20 min, followed by centrifugation at 10,000×*g* for 20 min. Added proteinase K according to the ratio of PRP:PBS:Protease K at 20:10:1, shake and mix, and let stand for 10 min at room temperature. Then, the precipitation reagent was added at 1/5 of the volume of PRP + PBS, and it was allowed to stand at 4 °C for 30 min. At room temperature, the mixture was centrifuged at 10,000×*g* for 5 min, the supernatant was aspirated, the pellet was resuspended by gently pipetting with PBS, and the resuspended exosomes solution was placed in a 1.5 ml EP tube and stored at − 80 °C. The protein content of PRP and PRP-exos was determined by bicinchoninic acid (BCA) protein assay kit (Beyotime, Shanghai, China) [27]. Protein concentrations of 5 μg/ml and 50 μg/ml were adopted to observe the effect on articular cartilage in vivo [28].

### Characterization and identification of PRP-exos

For exosomes obtained by extraction and purification, excessive freezing and thawing times should be avoided to avoid changes in the diameter and shape of exosomes, and electron microscopy should be performed as soon as possible. We dropped 15 μl PRP-exos onto the carrier copper mesh, let it stand at 20–25 °C for 2–3 min, removed excess liquid with filter paper on the edge of the copper mesh, dropped into uranyl acetate solution for staining for 2 min, removed the liquid with filter paper, and washed with PBS 1–2 times, the filter paper absorbs the liquid, and it was observed under the transmission electron microscopy (TEM) after drying at room temperature.

Particle Metrix ZetaView® nanoparticle tracking analysis (NTA) technology (Particle Metrix GmbH, Germany) was used to carefully estimate the size and concentration distribution of PRP-exos.

We added the protein quantified PRP-exos to an appropriate volume of protein loading buffer and mixed well with a shaker, heated at 95 °C for 4–7 min, and cooled it at room temperature to prepare protein samples. According to the concentration, we added about 30 μg protein amount of PRP-exos to the sample well of sodium dodecyl sulfate–polyacrylamide gel electrophoresis (SDS-PAGE) gel and added an appropriate amount of electrophoresis solution. Then, protein extracts were separated by 10% SDS-PAGE at 90 V for about 30 min. After electrophoresis, the gel was removed, and the edge was cut off and soaked in transfer buffer for about 10 min. We cut the PVDF membrane according to the size of the gel block, soak it in methanol solution for 10 s, and rinse it with deionized water 2–3 times. Put sponge pad, filter paper, PAGE gel, PVDF membrane, filter paper, and sponge pad in sequence in the clip, added an appropriate amount of membrane transfer solution and transfer membrane on ice. Immediately after the transfer, the protein membrane was placed in TBST and rinsed for about 3 min. The TBST washing solution was removed with filter paper, and the membrane was blocked in the blocking solution for 1 h. The membrane was probed with CD63 (1:1500 dilution, Wanleibio Co, Ltd. Shenyang, China), TSG101 (1:1000 dilution, Wanleibio Co, Ltd. Shenyang, China), and HSP70 (1:1000 dilution, Wanleibio Co, Ltd. Shenyang, China) overnight at 4 °C. After the primary antibody incubation, the protein membrane was soaked and washed with TBST washing solution (8–12 min each time, 3 times in total). Added the corresponding secondary antibody according to the species of the primary antibody, and left it at room temperature for 1 to 2 h. After the incubation, wash 3 times with TBST in the same step. Prepare the ECL fluorescent reagent (Thermo Fisher Scientific, Waltham, MA, USA) according to the instructions, drop it on the surface of the protein membrane, image it through the gel developing system, and perform imaging processing to obtain the imaging data.

### Preparation of modeling of cartilage defects in both knees and experimental groups

Anesthesia was administered by intraperitoneal injection of 3% pentobarbital sodium solution (Sigma-Aldrich) at a dosage of 1.0 ml/kg before the operation, the anesthesia was successful when the corneal reflex was sluggish and the pain stimulus response was significantly weakened. The skin below the abdomen was prepared and sterilized, and a sterile drape was placed. The skin was incised along the lateral side of the knee joint, the subcutaneous fascia was bluntly separated, the patellar ligament was exposed, the joint capsule was cut, the patella was dislocated medially, and the knee joint was flexed 90° to expose the femur condyle and patellofemoral articular surface, 2 mm grinding drill at low speed to prepare a full-thickness cartilage defect with a diameter of 2 mm and a depth of 2 mm, confirming that the depth reaches the subchondral bone plane (the grinding speed should not be too high during the modeling process to avoid the influence of high temperature and burning the surrounding bone and cartilage tissue are active, and if necessary, normal saline can be used for cooling treatment). After the modeling was completed, the joint cavity was washed with normal saline for several times, and the joint cavity and incision were infiltrated with penicillin for injection after no obvious bleeding. Finally, we stitched the joint capsule, subcutaneous fascia and skin, and covered the incision with sterile bandage. The contralateral knee joint was modeled in the same way.

According to the above method, we made models in 32 SD rats. Thirty-two SD rats were randomly divided into 4 groups: control group, PRP-exos (50 μg/ml) group, PRP-exos (5 μg/ml) group and PRP (50 μg/ml) group, 8 rats in each group. One week after operation, each rat was injected with PBS (control group), PRP-exos and PRP according to the grouping, 0.05 ml for each injection, once a week, twice in total. The PRP used for injection is extracted from fresh whole blood, stored at 4℃ and used within 2 h. And PRP was not activated, because platelets were activated when contacting with collagen tissue. Injection method: After the onset of anesthesia in the same way, the lower limbs of the rat were sterilized, the knee joint was straightened, the patella was slightly pushed to the outside after touching the patella, and the needle was inserted at the intersection of the upper edge of the patella and the outside. The resistance means that the puncture was successful, and the corresponding treatment was made according to the grouping conditions.

### Detection indicators

Four rats in each group were killed at the 5th and 10th week after treatment, respectively. The bilateral knee joints of each rat were collected, and the repair of cartilage defects was observed and scored. The results of HE staining and type II collagen immunohistochemical staining were included in the statistics. Before the rats were killed, we collected the serum of rats in each group and detected the levels of MMP-3 and TIMP-1 in serum by ELISA [29].

### Enzyme-linked immunosorbent assay

The anesthesia method was the same as above, the skin was cut on the inner side of the proximal femur, the tissue was bluntly separated, and the femoral vein was exposed. MMP-3 and TIMP-1 were measured by enzyme-linked immunosorbent assay (ELISA) kits from Jianglai Biotech according to the manufacturer's protocol. The plates were read at 450 nm. We set three wells for each experimental group.

### Tissue staining, immunohistochemistry analysis

The rat joints were fixed in 4% buffered paraformaldehyde for 24 h after the samples were harvested, decalcified in 10% (w/v) EDTA (pH 7.4) for 3 weeks at 4 °C, and embedded in paraffin. The samples were processed at 5 μm thickness by sagittal joint sections and stained with hematoxylin–eosin (HE). For each section, three high-power fields, containing the entire portion of the defects, were randomly sampled and the chondrocytes were counted. For IHC staining, the sections were rehydrated, treated to retrieve the antigen and incubated with the primary antibody at 4℃ overnight. Subsequently, the biotinylated secondary antibody and avidin–biotin-peroxidase complex were applied and the DAB substrate was used to visualize the stained sections. Then, the sections were counter-stained with hematoxylin, mounted and analyzed under a light microscope.

### Gross morphological observation and scoring of specimens

At the 5th and 10th weeks, 4 rats in each group were killed, and the bilateral knee joints were opened to observe the repair of cartilage defects and compare the repair conditions of each group. The scores were compared with the International Cartilage Repair Society (ICRS) [30]. A total of 3 people participated in the scoring of each sample, and the total score was included in the statistical test. The main observation contents and evaluation indexes include: the general condition of joint cavity, amount and nature of joint effusion, whether the new tissue is translucent, whether the surface is smooth, repair height, boundary fusion and overall abnormality assessment, etc.

### Statistical processing analysis

Statistical analysis was performed using GraphPad Prism software, and the data between the groups at the 5th week and the 10th week were compared. First, the normality test and the homogeneity of variance test were performed. According to the homogeneity or unequal variance, the independent sample means t test or t' test was used for comparison between groups, and the SNK-q test was used for pairwise multiple comparisons. *P* < 0.05 was considered to be statistically significant.

## Results

### Platelet-rich plasma extraction

Normal rat blood was tested for platelet count, the number was about 604.50*10^9^ pcs/L, and the number of platelets contained in PRP was about 2505.00*10^9^ pcs/L, which was 4.14 times that of normal whole blood (Table 1), which was in line with the platelet concentration of PRP Scope.

**Table 1:** Comparison of Platelet Numbers in Whole Blood and PRP (10^9^ pcs/L)

Number	Blood	PRP	Multiple
1	584	2371	4.06
2	638	2725	4.27
3	616	2588	4.20
4	569	2168	3.81
5	593	2391	4.03
6	627	2708	4.32
Mean ± SD	604.50 ± 26.733	2491.83 ± 219.08	4.12 ± .019

### Characterization of PRP-exos

**Fig. 1:**
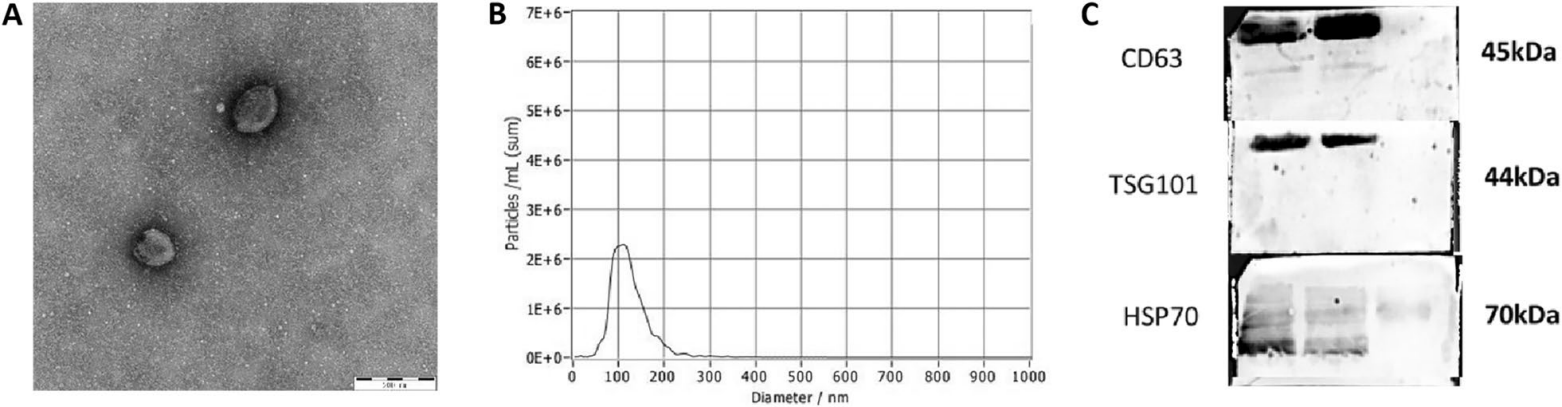
Characterization of PRP-exos: **A** Morphology observed by TEM. **B** Particle size distribution measured by NTA. **C** Western blotting and quantitative analysis of the exosomes surface markers and cargoes

The extracts observed under the TEM in this experiment were round and oval microvesicles with a size of 70–130 nm, which were cup-shaped and conform to the characteristics of exosomes under the electron microscope (Fig. 1a). By NTA detection, the diameter of the extracted PRP-exos was 123.05 ± 39.51 nm, and the concentration after 40 times dilution was 1.59 ± 0.72*10^6^ particles/ml, and the original sample concentration was 6.37 ± 2.89*10^7^ particles/ml (Fig. 1b). The extracted PRP-exos were analyzed by Western blot analysis. The characteristic proteins CD63, TSG101 and heat shock proteins 70 (HSP70) secreted by exosomes were all positive in PRP-exos, which further confirms that the extracts from this experiment were exosomes (Fig. 1c).

### General observation of cartilage repair

**Fig. 2:**
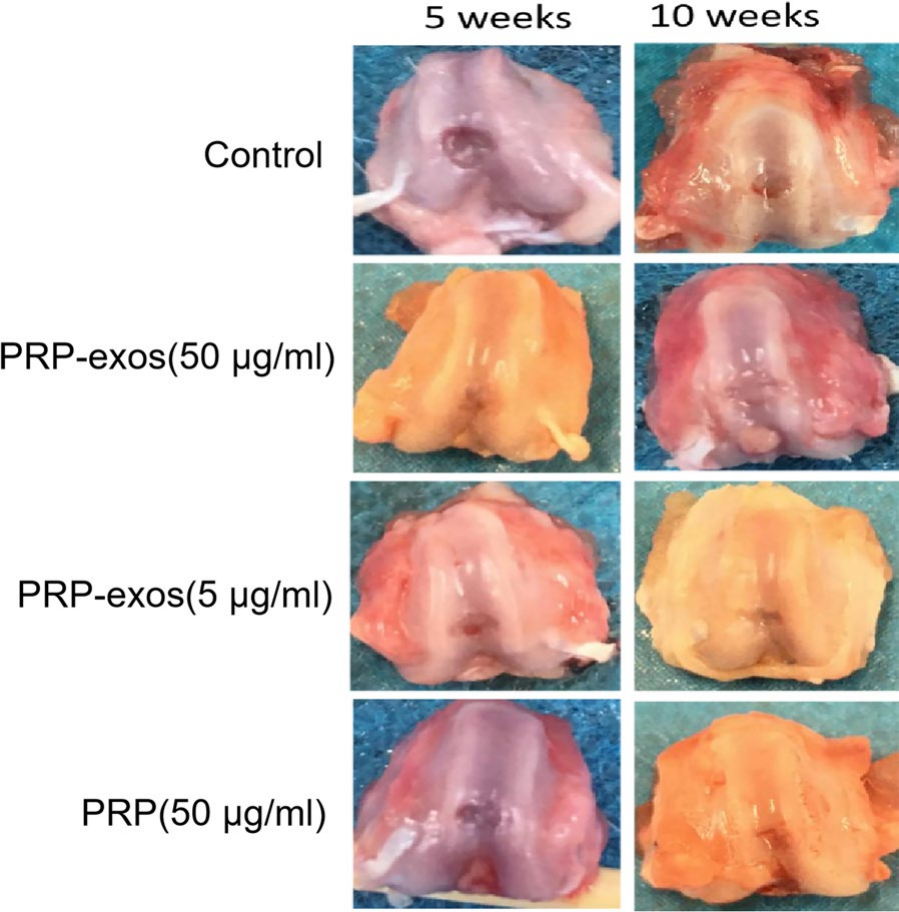
General observation of the repair of knee articular cartilage defect, left untreated control or treated with PRP-exos (50 μg/ml), PRP-exos (5 μg/ml) or PRP (50 μg/ml), at 5 and 10 weeks after operation

1. In the 5th week, the control group: a small amount of fluid in the joints, the nature of the fluid was slightly turbid, the synovial tissue was edema, there was no osteophytes or soft tissue hyperplasia around the joints, there was little new cartilage tissue, and the surface was opaque dark blue. It was not smooth, there were large cracks, the repair height was less than 25% of the original defect, and the defect boundary was poorly fused with normal tissue. PRP-exos (50 μg/ml) group: a small amount of effusion in the joint, the nature of the effusion was clear, the synovial tissue had no edema, there was no osteophyte or soft tissue hyperplasia around the joint, the new cartilage tissue was rich, the color was slightly translucent, the surface was not smooth, no cracks, and the repair was high—more than 75%, the defect boundary merges with normal tissue more than 3/4. PRP-exos (5 μg/ml) group: intra-articular effusion, the nature of the effusion was clear, the synovial tissue had no edema, there was no osteophyte or soft tissue hyperplasia around the joint, the new cartilage tissue was dark blue in color, opaque, fiber surface, scattered with small cracks, repair height was greater than 50%, and the fusion of the defect boundary with normal tissue was less than 1/2. PRP group: a small amount of fluid in the joint, the nature of the fluid was clear, the synovial tissue has no edema, there was no osteophyte or soft tissue hyperplasia around the joint, the color of the new cartilage tissue was slightly translucent, some of the colors was dark, the fiber surface was scattered with small cracks, and the height was repaired more than 50%, the defect boundary and normal tissue fusion were less than 1/2.
2. In the 10th week, the control group: a small amount of fluid in the joints, the nature of the fluid was slightly turbid, the synovial tissue was edema, there was no osteophytes or soft tissue hyperplasia around the joints, the color of the new cartilage tissue was not translucent, the surface was not smooth, scattered small for cracks, the repair height was greater than 50% of the original defect, and the defect boundary was poorly fused with normal tissue. PRP-exos (50 μg/ml) group: a small amount of fluid in the joint, the nature of the fluid was clear, the synovial tissue had no edema, there was no osteophyte or soft tissue hyperplasia around the joint, the new cartilage tissue was brighter, the surface was smooth, no cracks, and the repair height was greater than 75%, The boundary of the defect was basically fused with the normal tissue. PRP-exos (5 μg/ml) group: intra-articular effusion, the nature of the effusion was clear, the synovial tissue had no edema, there was no osteophyte or soft tissue hyperplasia around the joint, the color of the new cartilage tissue was slightly translucent, the fiber surface was scattered with small cracks, and the repair height was greater than 50%, the fusion of defect boundary with normal tissue was less than 1/2. PRP group: a small amount of fluid in the joint, the nature of the effusion was clear, the synovial tissue had no edema, the new cartilage tissue was slightly brighter, the fiber surface was scattered with small cracks, the repair height was greater than 50%, and the defect boundary and the normal tissue fusion were greater than 1/ 2 (Fig. 2).

### ICRS score

**Fig. 3:**
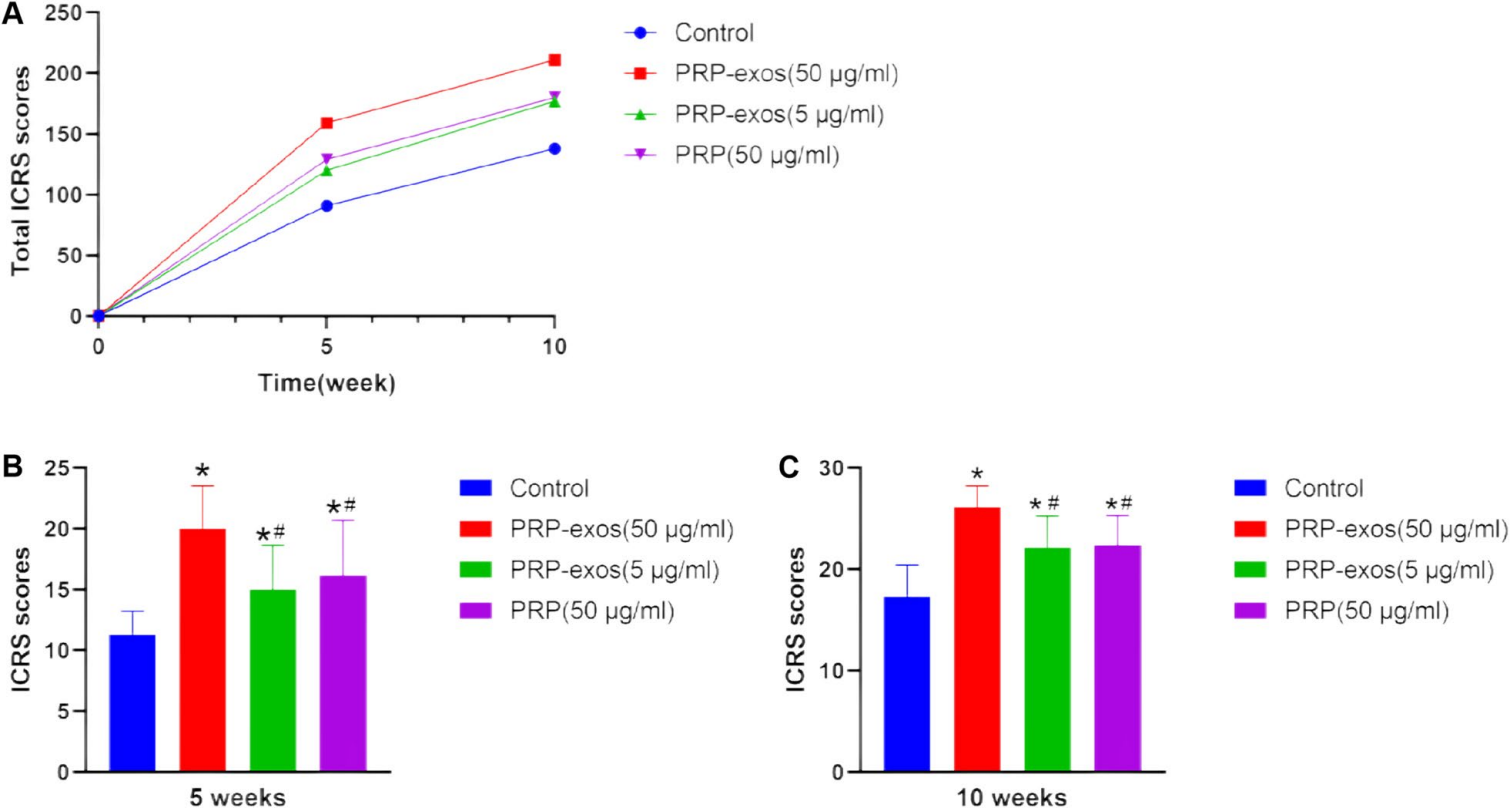
**A** Total ICRS scores of each group of specimens, **B** ICRS scores at 5 week, **C** ICRS scores at 10 weeks.*P < 0.05 compared to control group. #P < 0.05 compared with PRP-exos (50 μg/ml) group

The cartilage defect specimens in each group were scored by ICRS 3 times at the 5th and 10th weeks, respectively, and the total score of each group was calculated (Fig. 3a). The ICRS score of PRP-exos (50 μg/ml) group was always the highest, and the score of PRP group was slightly higher than that of PRP-exos (5 μg/ml) group, but the scores of the two were relatively close, and the control group had the lowest score. The multiple comparison results of each group showed that the ICRS score of each group at week 5 (Fig. 3b), PRP-exos (50 μg/ml) group had the most obvious advantage (*P* < 0.05), and PRP-exos (5 μg/ml) group and PRP group were better than the control group (*P* < 0.05), but there was no statistical difference between the PRP-exos (5 μg/ml) group and the PRP group. The ICRS scores of each group in the 10th week (Fig. 3c), PRP-exos (50 μg/ml) group had the most obvious advantage (*P* < 0.05), PRP-exos (5 μg/ml) and the PRP group were also better than the control group (*P* < 0.05), but there was no statistical difference between the PRP-exos (5 μg/ml) and the PRP group.

### HE staining results

**Fig. 4:**
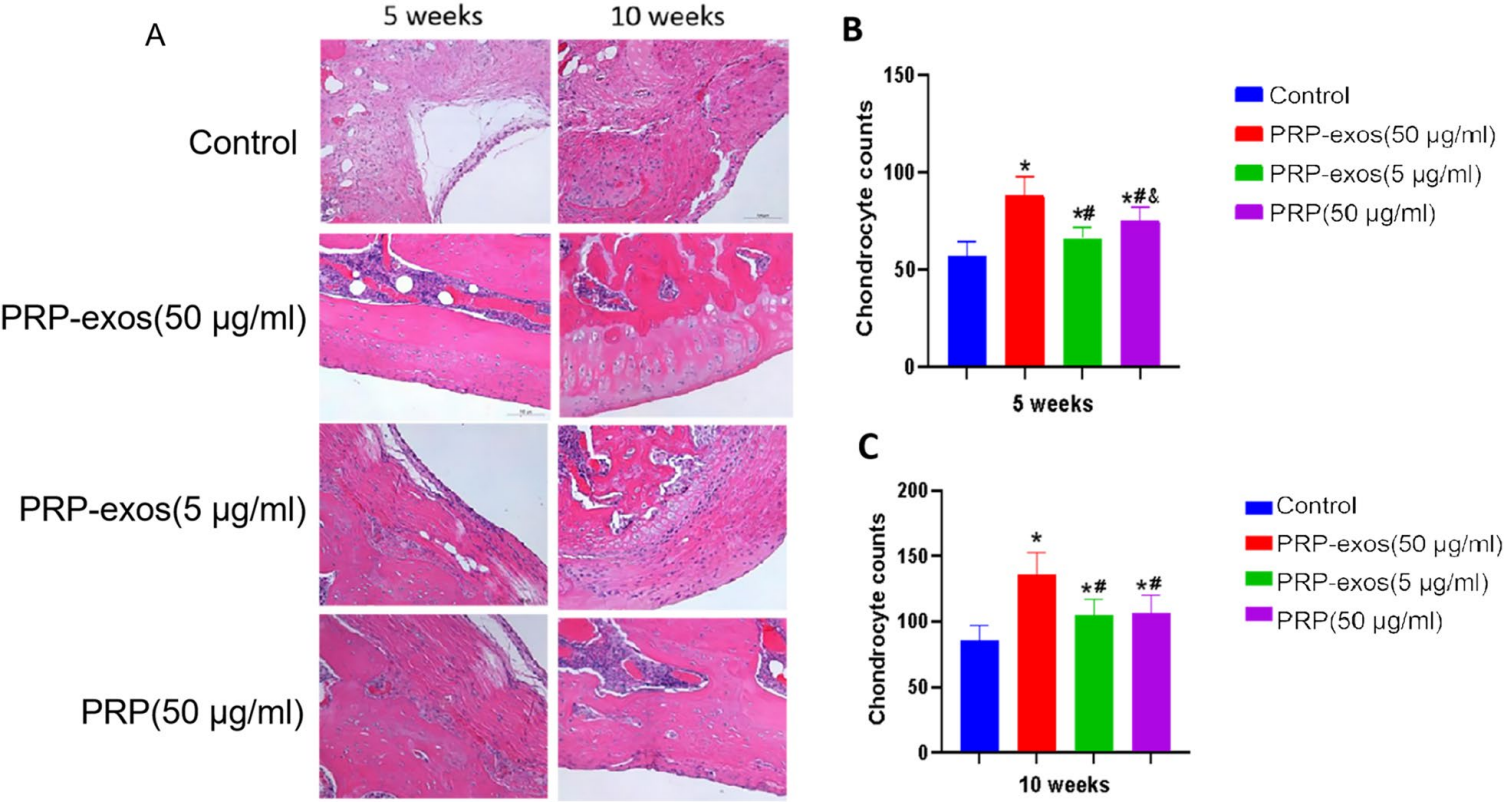
**A** HE staining results of cartilage defect repair tissue. Scale bar 100 μm, **B** chondrocyte counted at 5 weeks, **C** chondrocyte counted at 10 weeks. *P < 0.05 compared to control group. #P < 0.05 compared with PRP-exos (50 μg/ml) group. & P < 0.05 compared with PRP-exos (5 μg/ml) group

1. Normal articular cartilage tissue: the nucleus of chondrocytes was dark blue, the cytoplasm was transparent, the cartilage matrix was stained light blue, the surface of the cartilage membrane structure was visible, the superficial immature cells were smaller, the shape was elliptical, and the deep mature cells were larger and round. The clusters of homologous chondrocytes that gather together were arranged in a longitudinal direction perpendicular to the articular surface. The collagen fibers in the matrix were in an arched direction, parallel to the surface, and a longitudinal direction in the deep part.
2. In the 5th week, the control group: the number of chondrocytes was small, the arrangement was disordered, the fibrous proliferation was obvious but the shape was chaotic, the tissue structure was disordered, the surface was uneven, there were large defects and fissures, and local vascular proliferation; PRP-exos (50 μg/ml) group: chondrocytes were abundant, with larger and elliptical shapes. Some cells were arranged longitudinally, some fibrous proliferation, the tissue structure was more neat, the surface was relatively flat, and there were a few small fissures and local vascular proliferation; PRP-exos (5 μg/ml) group: more chondrocytes abundant, moderately sized, elliptical, disorderly arranged, fibrous hyperplasia was obvious, cartilage tissue structure was disordered, the surface was relatively flat, there were small fissures, and local vascular proliferation; PRP group: chondrocytes were more abundant, the shape was smaller and elliptical, the arrangement was disordered, the tissue structure was chaotic, the surface was relatively flat, there were small fissures, and local blood vessels were proliferated.
3. The 10th week, the control group: the number of chondrocytes was large, the arrangement was disorderly, the distribution is uneven, the fibrous proliferation was obvious, the trend was chaotic, the tissue structure was disordered, the surface was relatively flat, and there were still small fissures and local vascular proliferation; PRP-exos (50 μg/ml) group: the chondrocytes were dense, the superficial layer of immature cells were small, and the shape was elliptical or spindle, the deep layer of mature cells were larger and round, and the homogenous chondrocytes clustered together were arranged vertically to the articular surface, and a small amount fibrous hyperplasia, the fiber direction was arched, the tissue structure was relatively neat, the surface was flat, there were a few small fissures, and local blood vessels proliferation; PRP-exos (5 μg/ml) group: there were more cartilage cells, most of the cells are medium in size, elliptical, and some cells are arranged disorderly, fibrous hyperplasia was obvious, some of the tissue structure was disordered, the surface was relatively flat, there were small fissures, and local vascular hyperplasia; PRP group: chondrocytes were more abundant, the shape was small and elliptical, some cells were arranged in disorder, the fibrous hyperplasia was obvious, and the tissue structure was more orderly, the surface is relatively flat, there were small fissures, and local vascular hyperplasia (Fig. 4A).
4. Through the same magnification 40 times, counted the chondrocytes under the microscope several times, and make multiple comparisons between groups. In the 5th week, there are significant differences between the groups (*P* < 0.05). The number of chondrocytes in exosomes is the highest in PRP-exos (50 μg/ml) group, followed by PRP and PRP-exos (5 μg/ml) group. The control group is significantly less than the other three groups (Fig. 4b). In the 10th week, the number of chondrocytes is still the largest in PRP-exos (50 μg/ml) group, there is no statistical difference between the PRP group and PRP-exos (5 μg/ml) group (*P* > 0.05), and the number of cells in the control group is the smallest (Fig. 4c).

### Immunohistochemical staining results

**Fig. 5:**
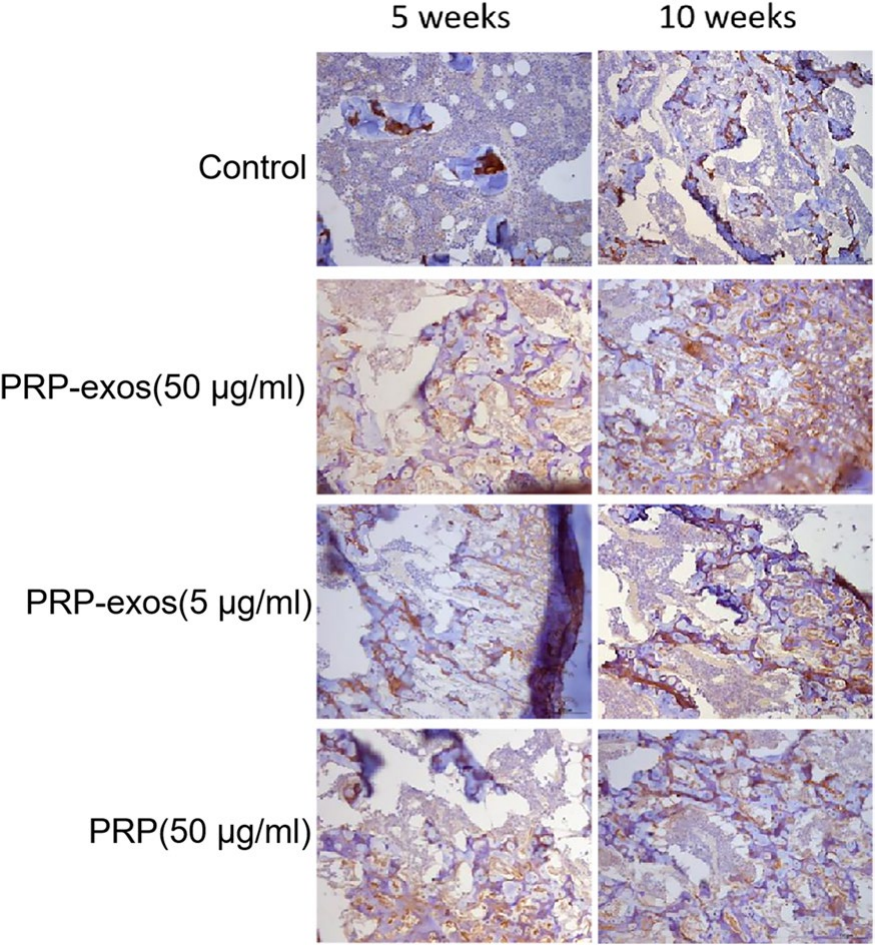
Type II collagen immunohistochemical staining. Scale bar: 100 μm

Through immunohistochemical staining, the more collagen fibers formed by type II collagen in the new cartilage tissue, the darker the brown color (Fig. 5).

1. In the 5th week, the control group: part of the cartilage matrix was light brown, and only a small area showed color, indicating that type II collagen fibers were less and unevenly distributed, and the tissue structure was chaotic; PRP-exos (50 μg/ml) group: The cartilage matrix was medium brown, and the color developing area was wide and evenly distributed, indicating that type II collagen fibers were rich and orderly; PRP-exos (5 μg/ml) group: the cartilage matrix was light brown, the color area was more limited but the color part was distributed even, indicating that there were fewer type II collagen fibers; PRP group: the cartilage matrix was light brown, and the color area was more limited and evenly distributed, indicating that there were fewer type II collagen fibers.
2. In the 10th week, the control group: the cartilage matrix was moderately brown with fewer color areas, suggesting that there were fewer type II collagen fibers and the organization structure was chaotic; PRP-exos (50 μg/ml) group: the cartilage matrix was darker brown, the coloring area was wide and evenly distributed, indicating that type II collagen fibers were abundant and the structure was neat and orderly; PRP-exos (5 μg/ml) group: the cartilage matrix was moderately brown, and the coloring area was wider but evenly distributed, indicating type II collagen fibers More, the organization structure was chaotic; PRP group: the cartilage matrix was moderately brown, the color area was wider and the distribution was not even, indicating that there were more type II collagen fibers and the structure was chaotic.

### Serum index results

**Fig. 6:**
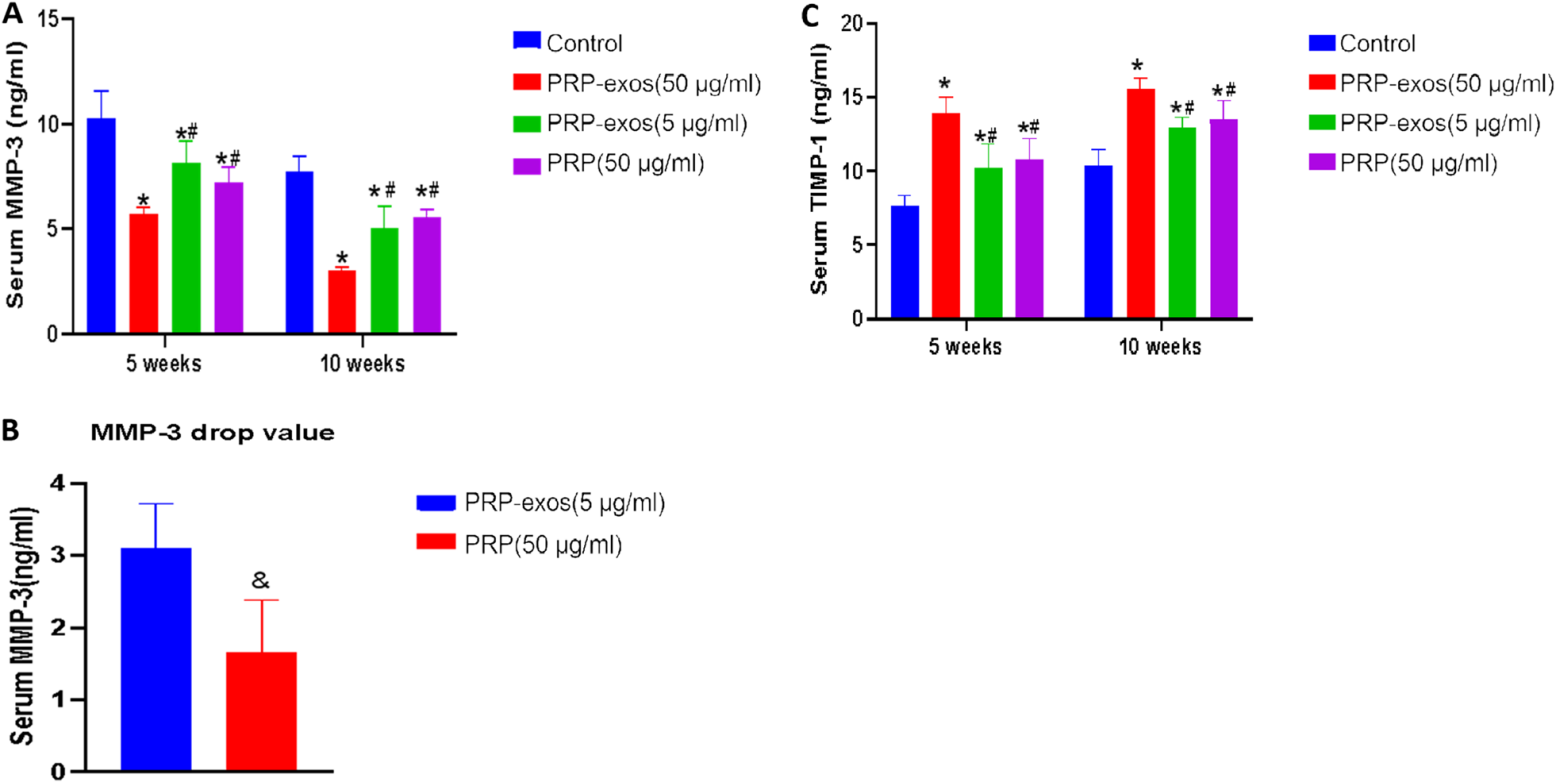
**A** Serum MMP-3 values in each group. **B** Decreased value of MMP3 in PRP-exos (5 μg/ml) group and PRP group in 5 weeks. **C** Serum TIMP-1 values in each group.*P < 0.05 compared to control group. #P < 0.05 compared with PRP-exos (50 μg/ml) group.& P < 0.05 compared with PRP-exos (5 μg/ml) group

1. Serum MMP-3 indicators: In the 5th week, the mean values of serum MMP-3 indicators in each group were PRP-exos (50 μg/ml) group (5.71 ng/ml), PRP group (7.21 ng/ml), PRP-exos (5 μg/ml) group (8.17 ng/ml) and the control group (10.27 ng/ml), through pairwise comparison between the groups, the serum MMP-3 index of PRP-exos (50 μg/ml) group was significantly lower than that of the other groups (P < 0.05). The PRP group was compared with exosomes. There was no significant difference in serum MMP-3 indicators between the two groups (*P* > 0.05), while the serum MMP-3 indicators in the control group were significantly higher than the other groups (*P* < 0.05). In the 10th week, the mean values of serum MMP-3 indicators in each group were PRP-exos (50 μg/ml) group (3.02 ng/ml), PRP-exos (5 μg/ml) group (5.07 ng/ml), PRP group (5.54 ng/ml) and control group (7.75 ng/ml), the statistical results were consistent with the 5th week (Fig. 6a). As the index of PRP-exos (5 μg/ml) group was higher in the 5th week than in the PRP group, but the index in the 10th week was lower than that of the PRP group, the serum MMP-3 drop values of the two groups were used for statistical comparison, and the t' test method was used. The results showed that the MMP-3 decrease value of PRP-exos (5 μg/ml) group was significantly higher than that of PRP group from week 5 to week 10 (*P* < 0.05) (Fig. 6b)
2. Serum TIMP-1 indicators: In the 5th week, the mean values of serum TIMP-1 indicators in each group were PRP-exos (50 μg/ml) group (13.87 ng/ml), PRP group (10.83 ng/ml), PRP-exos (5 μg/ml) group (10.21 ng/ml) and the control group (7.62 ng/ml), through pairwise comparisons between the groups, the serum TIMP-1 index of PRP-exos (50 μg/ml) group was significantly higher than that of the other groups (P < 0.05). The PRP group was compared with exosomes. There was no statistical difference between the two groups (P > 0.05). The index of the control group was significantly lower than that of the other groups (P < 0.05); at the 10th week, the mean serum TIMP-1 index of each group was in turn as that of PRP-exos (50 μg/ml) group (15.58 ng/ml), PRP group (13.51 ng/ml), PRP-exos (5 μg/ml) group (12.95 ng/ml) and control group (10.37 ng/ml), through pairwise comparison between the groups, the serum TIMP-1 index of PRP-exos (50 μg/ml) group. It was significantly higher than the other groups (*P* < 0.05). There was no statistical difference between the PRP group and the PRP-exos (5 μg/ml) group (*P* > 0.05). The index of the control group was significantly lower than the other groups (*P* < 0.05) (Fig. 6c).

## Discussion

In this experiment, based on the gross observation of cartilage defect specimens and ICRS score, the control group had synovial edema, turbid effusion, insignificant repair of the defect, large cracks, non-transparent tissue color, and poor fusion of the border with normal tissue. The remaining 3 groups had obvious defect repair, better transparency, and better fusion with normal tissue boundaries, indicating that PRP-exos and PRP are both beneficial to the repair of cartilage defects. Among them, 50 μg/ml PRP-exos has the best effect, indicating that it is more effective. High concentration of PRP-exos has a stronger repairing effect. In addition, 5 μg/ml PRP-exos and PRP showed no significant difference in general observation and ICRS score.

Hyaline cartilage has a high water content, so it is translucent to the naked eye. The fiber component is mainly type II collagen fibrils, which has strong pressure resistance and toughness. The immature cells in the superficial layer of articular cartilage are small and singularly distributed, while the deeper cells are larger, arranged in a single row vertical to the articular surface, and the fibers in the cartilage matrix are arched. Through the HE staining of specimen sections, the superficial naive chondrocytes, deep mature cells and homologous cell clusters in the PRP-exos (50 μg/ml) group can be seen under the microscope. The fibers in the matrix are arched, indicating that under the action of 50 μg/ml PRP-exos, not only the cartilage cells proliferate the most, but the tissue structure is also closest to the normal articular cartilage structure, which may have stronger pressure resistance, elasticity and toughness. Although the chondrocyte counts in PRP-exos (5 μg/ml) group and PRP group were better than those in the control group, the chondrocytes are relatively simple, the tissue structure is chaotic, and cartilage function may be poor.

After type II collagen IHC staining, the control group was stained lighter brown, with the smallest staining area and uneven distribution. The other three groups had wider staining areas and darker colors than the control group, indicating that the content of type II collagen was more, and the new tissue was hyaline cartilage.

The MMP family can degrade the extracellular matrix [31], and the TIMP family can inhibit this degradation [32]. The balance of the two can maintain the normal synthesis and catabolism balance of the extracellular matrix. Abnormal tissue stress, cartilage damage and other factors can induce the increased secretion of MMP-3, which in turn leads to excessive reduction of agglutinated glycans and fibronectin, which is not conducive to tissue repair. Among the serum MMP-3 indicators of each group, the PRP-exos (50 μg/ml) group, PRP group, and PRP-exos (5 μg/ml) group were lower than the control group, indicating that both PRP-exos and PRP can reduce the serum MMP-3 concentration, thereby reducing the decomposition and loss of cartilage matrix. In this experiment, we found that there was no significant difference in serum MMP-3 levels between PRP (50 μg/ml) group and PRP-exos (5 μg/ml) group at week 5 or week 10. However, we compared the decrease in serum MMP-3 value from 5 to 10 weeks, and the decrease in MMP-3 value in PRP-exos (5 μg/ml) group was better than that in PRP (50 μg/ml) group. On the other hand, the count of chondrocytes showed that at the fifth week, the count of chondrocytes in PRP (50 μg/ml) group was higher than that in PRP-exos (5 μg/ml) group. At the 10th week, there was no significant difference in chondrocyte count between PRP (50 μg/ml) group and PRP-exos (5 μg/ml) group. Therefore, we believe that although the protein concentration of PRP-exos (5 μg/ml) group is lower than that of PRP (50 μg/ml) group, the protection effect of PRP-exos (5 μg/ml) group on cartilage is stronger than that of PRP (50 μg/ml)group within 5 to 10 weeks. We speculate that the reason may be that the effect of PRP-exos is longer than that of PRP. Although the protein concentration of PRP-exos (5 μg/ml) group was lower than that of PRP (50 μg/ml) group.

TIMP-1 is a specific inhibitor of MMP-3, which protects cartilage tissue [33]. Comparing the serum TIMP-1 indexes of each group, the PRP-exos (50 μg/ml) group, PRP (50 μg/ml) group, and PRP-exos (5 μg/ml) group are all higher than the control group, indicating that both PRP-exos and PRP can increase the serum TIMP-1 concentration, thereby inhibiting MMP-3 degradation of cartilage tissue. Therefore, we believe that PRP-exos are beneficial to cartilage repair. In particular, higher concentrations of PRP-exos have a better protective effect on cartilage tissue.

There are still shortcomings in this experiment. The experiment uses allogeneic blood to extract PRP and PRP-exos. Although the rats used in the experiment are all homologous rats with high genetic similarity, the rejection is inevitable. In addition, due to the limited blood volume of rats, the PRP-exos used in the experiment were obtained by kit extraction instead of ultracentrifugation. It is not ruled out that the exosomes extracted by the two methods have differences in cartilage defect repair. Finally, in order to minimize the impact of rejection on PRP and PRP-exos in rats, we set up two groups of rats to extract PRP and PRP-exos, respectively, instead of collecting all rats' whole blood for homogenization and separating PRP and PRP-exos. However, different rats and different amounts of whole blood may contain different bioactive molecules. How to standardize the extraction of PRP-exos, which is worthy of further discussion in the future.

## Conclusion

In summary, intra-articular injection of PRP-exos and PRP can significantly promote the repair of articular cartilage defects in SD rats. And compared with PRP with protein concentration of 50 μg/ml, PRP-exos with protein concentration of 50 μg/ml have more advantages in promoting cartilage repair. The new cartilage tissue is closer in structure to the normal articular cartilage tissue and contains more type II collagen, so the cartilage tissue may have stronger compression resistance and toughness. In addition, the repair effect of PRP-exos has a certain correlation with the concentration, and the repair effect of the higher concentration of PRP-exos is better than that of the lower concentration of PRP-exos.

## Data Availability

Data will be available upon request by the first author HZ.

## Abbreviations

ELISA: Enzyme-linked immunosorbent assay
HE: Hematoxylin–eosin staining;
HSP: Heat shock proteins
ICRS: The international cartilage repair society
IHC: Immunohistochemistry
MMP-3: Matrix metalloproteinase-3
NTA: Nanoparticles Tracking Analysis
PBS: Phosphate buffer saline
PRP: Platelet-rich plasma
PRP-exos: Exosomes derived from platelet-rich plasma
TIMP-1: Tissue inhibitor of matrix metalloproteinases-1
TEM: Transmission electron microscope
TSG101: Tumor susceptibility gene-101
